# An unusual cause of chyluria after radiofrequency ablation of a renal cell carcinoma: a case report

**DOI:** 10.1186/1752-1947-5-307

**Published:** 2011-07-13

**Authors:** Tze Min Wah

**Affiliations:** 1 Clinical Radiology Department, St James's University Hospital, Leeds Teaching Hospitals Trust, Leeds, LS9 7TF, UK

## Abstract

**Introduction:**

This report highlights a rare cause of chyluria occurring after radiofrequency ablation of a renal cell carcinoma. The condition requires a high index of suspicion, as it may not be diagnosed routinely on imaging follow-up after treatment. As chyluria can vary from no symptoms to hypoproteinemia, hypolipidemia and impaired immune function, prompt diagnosis will allow timely management of symptoms.

**Case presentation:**

During a routine renal examination, an otherwise fit and well 79-year-old Caucasian man was found to have a peripherally situated tumor. He underwent renal radiofrequency ablation as primary treatment. Periodic imaging follow-up over two years showed no evidence of residual or recurrent disease within the zonal ablation. The routine imaging protocol at St James's Hospital included upper abdomen only for kidney assessment; pelvic examination was not included. However, our patient underwent a computed tomography scan of his abdomen and pelvis at the request of his local urologist, around two and a half years after the renal radiofrequency ablation. A fat-fluid level was seen within the urinary bladder, consistent with chyluria. As our patient was asymptomatic, he was treated conservatively.

**Conclusion:**

It is important to be aware of chyluria as a possible complication of renal radiofrequency ablation, and to recognize the fat-fluid level sign within the bladder or collecting system on computed tomography scans. As most institutions do not routinely perform computed tomography scans of the pelvis as part of their follow-up protocol after renal radiofrequency ablation, a high index of suspicion is required for diagnosis. Routine urine analysis for fat should be considered, as prompt diagnosis is crucial to guide management for symptomatic patients.

## Introduction

Image-guided percutaneous renal radiofrequency ablation (RFA) therapy is now an increasingly popular treatment for small and selected renal cell carcinoma. It has low morbidity, high technical success and good mid-term outcome results [1,2]. After renal RFA, treatment efficacy is usually monitored by periodic cross-sectional imaging, with either computed tomography (CT) or magnetic resonance imaging. Most institutions, especially in Europe, only perform cross-sectional imaging of the kidneys; the pelvis is not routinely imaged as part of the follow-up. I report a case of incidental diagnosis of chyluria on CT in a patient who had undergone renal RFA for a small left renal cell carcinoma. This case report highlights a rare but important cause of chyluria after renal RFA. Clinicians need to have a high index of suspicion, as this condition may not be diagnosed routinely on imaging follow-up. As chyluria can present with varying degrees of severity, prompt diagnosis would allow timely management of symptoms.

## Case presentation

A 79-year-old Caucasian man with benign prostatic hypertrophy underwent a routine renal ultrasound examination for lower urinary-tract symptoms. He was found to have a peripherally situated tumor 21 mm in size. He had no other relevant medical history and his serum creatinine was within normal limits (104 μmol/L). He was referred to my department for consideration of percutaneous renal RFA. This case was discussed at the local urology multidisciplinary meeting, where the consensus was to offer surgery such as radical or partial nephrectomy or percutaneous RFA. The treatment options and risks were discussed in detail with our patient, who agreed to proceed with percutaneous RFA as first-line treatment.

**Figure 1:**
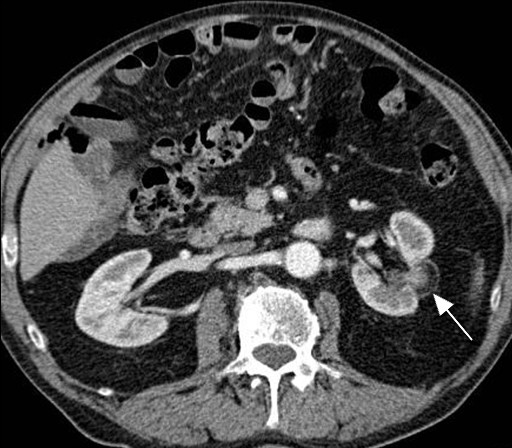
**Axial section of contrast-enhanced CT of his left kidney after RFA shows the treated region (white arrow) with no evidence of residual or recurrent disease at two years after RFA**.

**Figure 2:**
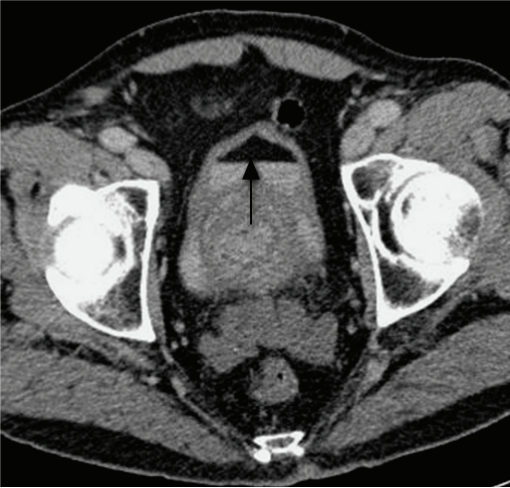
**Axial section of contrast-enhanced CT of the bladder shows a fat-fluid level (black arrow) within the bladder, consistent with chyluria**.

The procedure was performed under general anesthesia. A 16G co-access sheathed needle system (Boston Scientific, Boston, MA, USA) was inserted into the tumor under imaging guidance, using a combination of ultrasound and contrast-enhanced CT (100 mls Ultravist 300; Schering AG, Berlin, Germany). An 18G needle core biopsy of the tumor was obtained, immediately followed by the insertion of a Le Veen RFA array probe (Boston Scientific) with a diameter of 30 mm. Three overlapping treatments were performed, with a total ablation time of 20 minutes and 50 seconds. Our patient was given 80 mg gentamicin intravenously as prophylaxis. Histological examination of the tumor biopsy confirmed a grade 2 conventional renal cell carcinoma. Our patient was able to pass urine adequately after the procedure, and was discharged on the following day with stable renal function (creatinine 100 μmol/L). He was followed up with imaging at one, three, six and twelve months. Repeat imaging two years after the operation showed no evidence of residual of recurrent disease within the treated area (Figure 1). At St James's Hospital, the routine imaging protocol includes only the upper abdomen for kidney assessment; pelvic examination is not included. However, around six months later (about two and a half years after the surgery), our patient underwent a CT scan of his abdomen and pelvis at his local hospital at the request of his local urologist. A fat-fluid level was seen within the urinary bladder, consistent with chyluria (Figure 2). As he was asymptomatic, he was treated conservatively.

## Discussion

Chyluria is rare, and is caused by communication between the lymphatic and urinary tract systems. It is usually secondary to filariasis infection [3]. Other causes include abscesses, tumor, tuberculosis and congenital conditions. These are usually due to rupturing of the lymphatic system into the pelvicalyceal system. Rarely, iatrogenic causes of chyluria have been described after radical and partial nephrectomy, resulting in a fistulous connection from the lymphatics to the collecting system [4-6]. Interestingly, two cases of asymptomatic chyluria were recently diagnosed during routine follow-up after renal RFA in California, USA, where the routine imaging included both the abdomen and pelvis [7].

Renal RFA has been in practice for over 10 years and it is interesting to note that many complications related to the procedure have been well reported but, until recently, chyluria was not one of these. Because my hospital does not usually include pelvic examination during follow-up, it is likely that the diagnosis of asymptomatic chyluria after renal RFA will be missed unless the patient is symptomatic. In the case described here, the diagnosis only came to light when our patient's pelvis was scanned for other clinical reasons. The lack of reports of chyluria after renal RFA to date could be related to the fact that most follow-up protocols do not routinely include examination of the pelvis. However, it is extremely important to recognize the fat-fluid level sign in the bladder or collecting system on CT, especially in patients with a previous history of renal ablation. Early recognition would allow prompt diagnosis of chyluria. As chyluria can vary from no symptoms to hypoproteinemia, hypolipidemia and impaired immune function, prompt diagnosis would allow timely management of symptoms [8]. Usually, symptomatic patients report milky-white urine, and fat can be detected on urine analysis. Treatments include nutritional support, renal sclerotherapy and surgical ligation of the lymphatic system [4,9].

## Conclusion

It is important to be aware of chyluria as a complication after renal RFA, and to recognize the fat-fluid level sign within the bladder or collecting system on CT. As most institutions do not routinely perform CT of the pelvis as part of their follow-up protocol after renal RFA, a high index of suspicion is required for diagnosis. Routine urine analysis for fat should be considered in such patients, as prompt diagnosis is crucial to guide management.

## Consent

Written informed consent was obtained from the patient for publication of this case report and accompanying images. A copy of the written consent is available for review by the Editor-in-Chief of this journal.

## Competing interests

The author declares that they have no competing interests.
